# Incompletely Evaluated ART Leading to Ectopic Pregnancy and Cerebral Thrombosis

**Published:** 2013-07-31

**Authors:** Hikmet Hassa, Yunus Aydin, Tufan Oge, Vehbi Yavuz Tokgoz

**Affiliations:** Department of Obstetrics and Gynecology, Reproductive Medicine Unit, Medical Faculty, Eskisehir Osmangazi University, Eskisehir, Turkey

**Keywords:** Assisted Reproductive Technology, Thrombosis, Ectopic Pregnancy

## Abstract

We presented a cerebral venous thrombosis case associated with lack of proper medical evaluation required for confirmation of suppression and exclusion of current pregnancy before starting assisted reproductive technology (ART) cycle. This is a case-report study about a 37-year-old woman who presented to emergency room with abdominal pain and tenderness. Initial human chorionic gonadotropin (hCG) value was 17616 IU/L. Endometrium was heteregenous and incompatible with a normal intrauterine pregnancy. She had a history of antagonist protocol/controlled ovarian hyperstimulation (COH) started 38 days ago in a different *in vitro* fertilization (IVF) center. Because of the fertilization failure, she had no embryo transfer. With ectopic pregnancy diagnosis, we made surgical exploration and observed a material which was consistent with ectopic pregnancy on the right tuba uterina. Partial salpingectomy was applied because of actively bleeding ectopic pregnancy. Two days after discharging from hospital; she presented to emergency room again with syncope and generalized tonic-clonic seizure. By cranial tomography generalized edema, cerebral venous thrombosis was established. Enoxaparine sodium 0.6 ml twice daily was administered. Six days after hospitalisation, she was discharged with normal neurological examination under phenytoin 200 mg daily and enoxaparine sodium 0.6 ml daily. Before ART treatment, clinicians must always rule out the likelihood of existing pregnancy by measuring estradiol, follicle stimulating hormone (FSH), and luteinizing hormone (LH). On the other hand, low-molecularweight heparine may be effective in cerebral venous thrombosis treatment. Therefore, intracerebral thrombosis is one of the rare mortal complications of ART.

## Introduction

General principles in the evaluation of infertile couple are almost universal, including midluteal phase progesterone assay, semen analysis and a test for tubal patency such as hysterosalphingography. Before starting the treatment, especially in the form of assisted reproduction technology (ART), existing pregnancy must be ruled out (1). Since its development over 25 years ago, ART is widely practiced. However, many complications may develop as a consequence of ART. Beside the ovarian hyperstimulation syndrome (OHSS) as the most frequent one, venous thrombo-embolism (VTE) is one of the most devastating complication of ART. VTE (75%) is commonly found more than arterial thrombo-embolism (25%) in patients following ART (2). Intracerebral thrombosis may end up with stroke and is mostly associated with OHSS in patients receiving ART treatment. Thrombosis of the dural sinus and/or cerebral veins (CVT) is an uncommon form of stroke, representing only 0.5 to 1% of this incident (3).

We are presenting a cerebral venous thrombosis case associated with lack of proper medical evaluation required for confirmation of suppression and exclusion of current pregnancy before starting ART cycle.

## Case Report

In this case-report study, a 37-year old woman presented to our emergency room with abdominal pain. Her pulse rate was 72 beats per minute, and blood pressure was 80/50 mmHg. We revealed abdominal tenderness, muscular defence and rebound tenderness on her pelvic examination. In initial, complete blood count analysis hemoglobin level was 7.9 g/dL, hematocrit level was 31% and platelet count was 177×10^3^/μl. Initial human chorionic gonadotropin (hCG) value was 17616 IU/L. With ultrasound examination, we determined 4×2 cm cystic structure at right adnexial region, which is compatible with corpus luteum and bilaterally enlarged ovaries. Additionally, endometrium was heterogenous, but it was not compatible with a normal intrauterine pregnancy with a hCG value as 17616 IU/L.

According to knowledge from patient’s history, 38 days ago, on the second day of her menstrul cycle, she had admitted to another infertility clinic with secondary infertility (unexplained). Before starting ART therapy, only basal transvaginal- ultrasonography (TV-USG) had been performed, while basal hormone profile, the thickness of endometrium and antral follicle count had not been measured. We also learned from patient that on her first vist of the center, on the second day of her menstrul cycle, controlled ovarian stimulation had been started with 375 IU doses of gonadotrophin. Treatment had been continued with constant doses. On the sixth day of the stimulation, GnRH antagonist-Cetrorelix 0.25 mg/day had been started. On the twelfth day of the stimulation, 250 mcg hCG had been administered. Transvaginal oocyte retrieval had been performed on the fourteenth day of the stimulation, and one oocyte had been yielded. Sixteen hours after intracytoplasmic sperm injection (ICSI), since oocyte had not been fertilized, embryo transfer did not happen.

After the first evaluation in emergency room, we took the patient to the operation room with indication of surgical exploration of abdomen for suspicion of ectopic pregnancy. In the exploration, we observed a material which was consistent with ectopic pregnancy within hemorrhagic ruptured region of the right tuba uterina. Partial salpingectomy was applied because of actively bleeding ectopic pregnancy. We made sequential measurement of hCG, so on first and fifth postoperative days, hCG values were 4563 IU/L and 1200 IU/L, respectively. Patient was discharged from hospital without any complaints with suggestions for hCG follow-up. Pathologic result of the material was 'immature chorion villus and desidual tissue', being consistent with ectopic pregnancy.

Two days after hospital discharge, she presented to emergency room again with syncope and generalized tonic-clonic seizure. On neurological examination, she showed apathy and no lateralization. Generalized edema and suspicious cerebral venous thrombosis were determined on the cranial computerized tomography. She was admitted to the neurology department. On complete blood counts, white blood cells were in normal range, while hemoglobin (9.9 g/dl) and hematocrit (29.8%) were slightly decreased. Platelet counts were normal (273×10^3^/μl). Serum concentrations of electrolyte were also in normal range.

Following hospitalization, she had generalized tonic-clonic seizures with half hour intervals. Rapidly enoxaparine sodium 0.6 ml twice daily, midazolam infusion, and phenytoin sodium 100 mg twice daily were administered. She was also conservatively treated with balanced electrolyte solution. The result of screening test for antithrombin III, Leiden V factor, protein C and S deficiency, antinuclear antibodies (ANA), Anti-DNA, lupus anticoagulant and ENA panels were negative. After recovery, venous phase magnetic resonance (MR) angiography was performed. MR angiography demonstrated irregular filling on the superior sagittal sinus. The patient was assessed as cerebral venous thrombosis with these findings. Her neurologic status was stabilized after a few days. Six days after hospitalisation, she was discharged with normal neurological examination under phenytoin 200 mg daily and enoxaparine sodium 0.6 ml daily. For two consecutive months after discharge from the hospital, we monitored the patient with routine neurological and gynecological examinations.

## Discussion

Essential adaptation of mother’s hemostatic system to pregnancy is generally protective for peripartum unwanted bleeding, but also contributes to increased risk of thromboembolic complications. Venous thromboembolism is a rare disorder occurring in approximately 1/1000 to 1/2000 pregnancies, which can lead to serious mortality and morbidity in pregnant women (4). Thromboembolic disease is the leading cause of maternal mortality, especially in developed countries (5). Many of the physiological changes of pregnancy predisposes the thrombosis by contributing the classical Virchow’s triad as stasis of blood, hypercoagulability and vascular changes (6).

One of the most devastating result of thromboembolism during pregnancy is cerebrovascular event due to thrombosis of dural sinuses, cerebral veins or arteries. The overall risk of pregnancy- related stroke was generally 34 in 100,000 deliveries (7). Beside the pregnancy, other related risk factors are hypertension, preeclampsia, infection, delivery, the puerperium, nonwhite race and age older than 35. In addition, common primary conditions are antithrombin III, protein C, protein S deficiency, as well as the presence of antiphospholipid antibodies, anticardiolipin antibodies, factor V leiden gene mutation, resistance to activated protein C, and prothrombin G20210A mutation (3,8).

It is evident that the increas in level of estradiol accompanying ART treatment is one of the most important factors for complications as OHSS or thromboembolism. In addition, increase in the levels of both coagulation factors (such as Von Willebrand factor (vWF), factors VIII and V, and fibrinogen) and protein C-S activity, while decrease in antithrombin-III (AT-III) during controlled ovarian hyperstimulation are responsible for complications. Venous thromboembolism (75%) is commonly found more than arterial thromboembolism (25%) in following ovarian stimulation for ART, and also it is shown in up to 95% of cases associated with OHSS (2). Following ART, arterial thrombosis develops mostly in intracerebral vessels, while venous thrombosis occurs mostly in uncommon sites, such as upper limb, head and neck veins. Cerebral sinus thrombosis are mostly unusual following ART treatment in infertile patients; therefore, there are a few cases with cerebral sinus thrombosis in literature.

With the advancement of new perspectives in ART, evaluation of the infertile couple also changed. Today, a widely accepted approach to infertility does not include the diagnosis of an exact etiology. The scope and sequence of the modern infertility evaluation have shifted focus, from making a speciﬁc diagnosis to using the most efﬁcient and cost-effective tests. It is now obvious that investigation of infertile couple should be concluded rapidly and inexpensively with least invasive tests (1). As a result, ART has been applied either to some incompletely investigated couples which leades to some complications, as in our case, or to unnecessary over-treatment of some subfertile couples. According to recorded information obtained from the first infertility clinic, patient’s protocol/controlled ovarian hyperstimulation (COH) treatment had been started with vaginal bleeding, which was assumed to be the starting of menstrual cycle, so the blood estrodiol level was not checked. It is evident that they had not followed the guidelines for suppression or absence of an existing pregnancy before starting COH treatment (9,10). It can be explained that they had not obtained sufficient mature follicles, and subsequently, no embryos for transfer because most probably, they had started the cycle with the vaginal bleeding caused by ectopic pregnancy.

Our case had two major risk factors, including: i. delayed diagnosis of existing ectopic pregnancy due to absence of vaginal bleeding from high estradiol level resulted in tubal rupture and ii. cerebral venous thrombosis. Inherited or acquired thrombophilia was not detected in our case. Although syndrome of ovarian hyperstimulation was reported in 90% of all cases involving arterial thrombosis and in 78% of women with venous thrombosis, it was not a mandatory predisposing factor in our patient because she did not show any sign of OHSS (11). Clinical process of patient was most probably caused by erroneously started COH cycle and/or ectopic pregnancy because starting COH cycle with insufficient control masked the existing ectopic pregnancy. As recored in the first evaluation of the case in our clinic, she had a hCG value as 17616 IU/L. It is noted that low molecular weight heparine is effective even in the presence of two dominant predisposing factors (existing ectopic pregnancy and ART) for thromboembolism as in our patient. So, the main aim must be starting anticoagulant treatment as soon as possible in the presence of suspicion (12).

Therefore, this is a unique case with a history of COH treatment with insufficient control masked the existing ectopic pregnancy, and was also complicated with cerebral thrombosis. ART became a rather frequently administered treatment option for infertile couple in recent years, but clinicians must always bear in mind complications of ART. Therefore, for prevention of any complications, the major steps in evaluation of infertile couple should be taken in order to prove the suppression or to rule out the possibility of pregnancy before starting COH cycle.
